# Nonrelativistic Spin Current Generated Unconventional Spin Hall Magnetoresistance in Altermagnetic RuO_2_

**DOI:** 10.1002/advs.202521779

**Published:** 2026-03-18

**Authors:** Chang Pan, Shuai Hu, Fanli Yang, Dongchao Yang, Wei Zhu, Jianwei Zhang, Weijia Fan, Zhong Shi, Liqing Pan, Shiming Zhou, Xuepeng Qiu

**Affiliations:** ^1^ School of Physics Science and Engineering Tongji University Shanghai China; ^2^ Institute of Quantum Materials and Devices School of Electronic and Information Engineering Tiangong University Tianjin China; ^3^ Hubei Engineering Research Center of Weak Magnetic‐Field Detection College of Science China Three Gorges University Yichang China

**Keywords:** altermagnet, spin currents, spin splitting

## Abstract

The intrinsic spin‐split band structure and compensated magnetic moments of altermagnets make them promising candidates for spintronic applications. Recently, a magnetic spin Hall effect has been discovered in spin‐split antiferromagnets, including noncollinear antiferromagnets and altermagnets. This effect enables the efficient generation of unconventional spin currents even without spin‐orbit coupling. However, although such nonrelativistic spin currents are proposed to have a magnetic origin, the direct connection between the Néel vector and the spin current polarization is still missing. Here, we report an unconventional spin Hall magnetoresistance (SMR) generated from nonrelativistic spin currents, which exhibits a fundamentally distinct angular‐ and temperature‐dependences compared to conventional SMR. Based on this mechanism, we investigate RuO_2_, an altermagnetic candidate under debate recently, and unveil its spin current polarization by disentangling the conventional and magnetic spin Hall effects. The results suggest that the nonrelativistic spin current hosts a polarization very close to the Néel vector, unambiguously indicating a magnetic origin. The unconventional SMR not only presents solid evidence helpful to solve the puzzle of state in RuO_2_, but also paves a straightforward route to harnessing nonrelativistic spin currents in altermagnets for advanced spintronic applications.

## Graphical abstract

It is reported that the unconventional spin Hall magnetoresistance generated from nonrelativistic spin currents in RuO_2_ is capable of accurately detecting spin current polarization and disentangling the $\hat{T}$‐odd and $\hat{T}$‐even components of a spin current. This work unambiguously reveals the magnetic origin of the nonrelativistic spin current in RuO_2_, and paves a straightforward route to understand the unconventional spin currents that are crucial in spintronics.

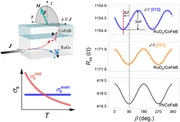

## 1 Introduction

Spintronics exploits the spin degree of freedom in electronic devices for information processing and storage [1]. The magnetic order parameters of magnetic materials serve as the state variables in spintronic devices, making the manipulation of magnetic states essential for the write‐in operation. “Standard” method for this sake relies on a magnetic field, which suffers a significant energy dissipation. Recently, it was found that the electrically generated spin currents can exert spin torques in magnets [2, 3, 4, 5, 6, 7, 8, 9, 10, 11, 12], providing an effective way of manipulating the magnetization in spintronic devices.

Conventionally, there are two approaches for generating spin currents and the associated spin torques. The first one utilizes a current perpendicular‐to‐plane (CPP) device, such as a magnetic tunnel junction (MTJ), where a longitudinal spin current from a ferromagnetic electrode tunnels across the barrier and transfers the spins toward another electrode [13, 14, 15]. Such a spin current is polarized by the spin‐split Fermi surface of the ferromagnetic electrode, and hence is collinear to the magnetization. The resultant spin‐transfer torque (STT) directly competes with the damping of the magnetization, leading to an efficient switching [15]. This approach, however, requires a substantial incubation time to initiate the torque dynamics, lowering the switching speed [16, 17]. Another approach employs the phenomena associated with spin‐orbit coupling (SOC), such as spin Hall effect (SHE) [10, 18] and Rashba‐Edelstein effect (REE) [9, 19, 20], generating a transverse spin current from a spin source layer or an interface and exerting the torques on the adjacent magnetic layer. Although such spin‐orbit torque (SOT) in the associated current in‐plane (CIP) devices shows the advantages such as the absence of read–write–breakdown voltage interferences, the difficulty of realizing a deterministic switching of perpendicular magnetization limits its application [9, 10, 21].

Recent findings have shown that some antiferromagnets with unconventional magnetism [22], including the noncollinear antiferromagnets and altermagnets, host momentum‐dependent spin splitting even in the absence of SOC [23, 24, 25, 26, 27, 28, 29, 30, 31, 32, 33, 34, 35, 36, 37, 38, 39]. The anisotropic spin distribution on the Fermi surface of these spin‐split antiferromagnets results in unconventional transverse spin currents [23, 28, 40, 41, 42, 43, 44, 45, 46, 47, 48, 49, 50]. This nonrelativistic spin current is time‐reversal‐odd and can be reversed by switching the Néel vector [23, 40, 48]. Such a phenomenon has been dubbed magnetic spin Hall effect (MSHE) [23, 41] or time‐reversal‐odd ($\hat{T}$‐odd) [49], to distinguish it from the conventional time‐reversal‐even ($\hat{T}$‐even) SHE due to SOC. Since the $\hat{T}$‐odd nonrelativistic spin current depends on antiferromagnetic spin texture such as Néel vector, it hosts unconventional spin polarization incapable of the $\hat{T}$‐even spin current, and hence can be used to realize efficient and deterministic switching in CIP spin torque devices [42, 44, 49].

So far, the unconventional spin polarization of the $\hat{T}$‐odd spin currents have been observed in many split antiferromagnets, including Mn_3_AN (A = Ga, Sn) [42, 51], Mn_3_X (X = Sn, Pt) [44, 45, 52], and RuO_2_ [48, 49, 50, 53, 54, 55]. However, the existing experiments are not sufficient to connect the spin current polarizations to the spin texture such as the Néel vector of these antiferromagnets. One possible reason is that the $\hat{T}$‐odd and $\hat{T}$‐even spin currents are intertwined [45] and hence a real polarization of the $\hat{T}$‐odd spin current is difficult to derive. A typical example is rutile RuO_2_, a canonical candidate of recently proposed altermagnet, which is widely employed to demonstrate spin‐dependent transport properties such as $\hat{T}$ symmetry breaking in the band structure [56], anomalous Hall effect [45, 57, 58], unconventional spin current [40, 48, 49, 50, 59], tunneling magnetoresistance [60, 61, 62, 63], and spin‐transfer torque [61] in antiferromagnetic tunnel junctions [64]. Especially, it was predicted to host notable $\hat{T}$‐odd spin current through the spin splitting effect (SSE) [40], hereafter referred to as the SSE spin current, and both the conventional component $\sigma_{zx}^{y,\mathrm{SSE}}$ and the unconventional $\sigma_{zx}^{x,\mathrm{SSE}}$ and $\sigma_{zx}^{z,\mathrm{SSE}}$ of spin conductivity have been found in low symmetric orientated (101)‐ and (110)‐oriented RuO_2_ films [48, 49, 50], which are considered to be associated with the SSE spin current. Recently, several theoretical and experimental [65, 66, 67, 68] works suggest a nonmagnetic ground state of RuO_2_, while other works [40, 48, 49, 50, 56, 57, 59, 62, 64] support its magnetic nature, bringing further questions about the origins of the above‐mentioned spin‐dependent transport properties.

As a fingerprint phenomenon of altermagnetism, the SSE spin current is expected to be strong and polarized along the Néel vector in an altermagnet, and hence the accurate detection of the spin current polarization of RuO_2_ would offer unambiguous evidence of its magnetic ground state. However, the spin current polarization derived based on the commonly used measurement is indeed tilted, but not consistent with the assumed Néel vector direction. This is possibly due to that the conventional SHE‐induced spin current, hereafter referred to as the SHE spin current, might be not negligible in contrast to the expectation. As a result, the intertwined SSE and SHE spin currents lead to an anomalous spin polarization [45] away from the Néel vector. A new method capable of disentangling the SSE and SHE spin currents is thus required to solve the problem.

Here, we demonstrate an unconventional spin Hall magnetoresistance (SMR) emerging from nonrelativistic spin currents which enables quantitative detection of unconventional spin current polarization and apply it to RuO_2_. Based on the measurement of unconventional angular‐ and temperature‐dependences of SMR in a RuO_2_/CoFeB heterostructure, we disentangle the spin conductivities induced by SSE and conventional SHE (hereafter referred to as the SSE and SHE spin conductivities) and derive the associated spin current polarizations. The results suggest the SSE spin current in RuO_2_ is dominant and hosts the polarization very close to the Néel vector direction at low temperature, indicating its magnetic origin. We also find the conventional SHE spin current is very strong and is dominant at high temperature.

## 2 Results and Discussion

**FIGURE 1:**
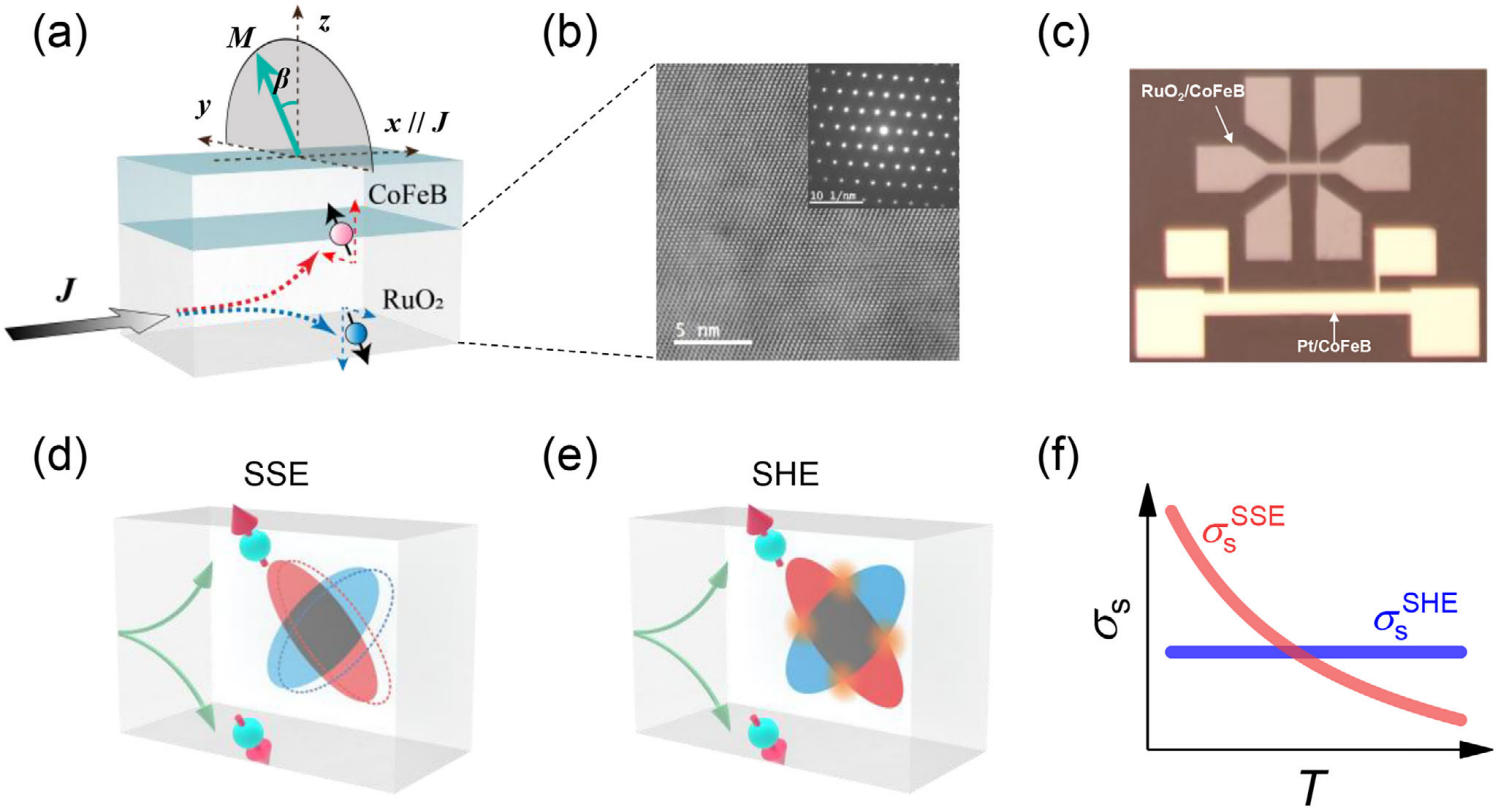
(a) SMR measurement scheme of RuO_2_/CoFeB heterostructure: magnetoresistance measured while scanning a 4 T magnetic field in a plane perpendicular to the electric field, and *β* is the angle between the magnetic field and ***z***. (b) Cross‐sectional scanning transmission electron microscopy (STEM) image of RuO_2_ (101). Inset: fast Fourier transform (FFT) of the STEM image. (c) Optical image of the RuO_2_/CoFeB (upper) and Pt/CoFeB (lower) device used for SMR measurements, in the former current direction along RuO_2_[010] and current in the latter is parallel to the former. The channel width of RuO_2_/CoFeB device is 16 µm. (d,e) Schematics of charge‐spin conversion in altermagnetic RuO_2_ due to nonrelativistic spin splitting (d) and conventional spin Hall effect (e). *N* indicates the Néel vector in (d). (f) A schematic of temperature dependences of SSE and SHE spin conductivities.

In this work, (101)‐oriented RuO_2_ and amorphous CoFeB on Al_2_O_3_ (1$\bar{1}$02) substrates have been prepared. Here, the current is along one specific crystallographic direction, and the current direction was defined as ***x***, and the normal direction as ***z***. During the measurement, a 4 T external magnetic field was rotated in the ***y***–***z*** plane, as illustrated in Figure 1a, while a 100 µA DC current was applied along the ***x*** direction and the longitudinal magnetoresistance (*R*) was measured using the four‐point method. Figure 1b is the cross‐sectional scanning transmission electron microscopy (STEM) image showing the epitaxial growth of high quality of RuO_2_ (101). The microscopy image of our devices is shown in Figure 1c, including both the RuO_2_/CoFeB (upper) and Pt/CoFeB (lower) reference devices.

The SSE and SHE spin currents arise from different origins. In a collinear magnetic metal under the nonrelativistic limit, the conductivity σ_*ba*_ can be decomposed into those contributions from the spin‐up (↑) and spin‐down (↓) Fermi surfaces, expressed as $\sigma_{ba}=\sigma_{ba}^{↑}+\sigma_{ba}^{↓}$, where *a* and *b* are the applied electric field and the generated charge current directions, respectively. The SSE spin conductivity is then $\sigma_{ba}^{s,SSE}=\sigma_{ba}^{↑}\;-\sigma_{ba}^{↓}$ (Figure 1d), where the spin polarization ***s*** is parallel to the magnetic order parameter [40]. On the other hand, the $\hat{T}$‐even spin conductivity $\sigma_{ba}^{s,\mathrm{SHE}}$ is associated with SOC and contributed by the spin‐Berry curvature within the band gaps [18], where ***s*** is determined by the crystal symmetry operations (Figure 1e). Since exchange splitting is usually much stronger than SOC, the SSE spin current is expected to be much stronger than the $\hat{T}$‐even one. Since $\sigma_{ba}^{s,\mathrm{SSE}}$ and σ_*ba*_ are strongly related, they should have the same temperature dependence, i.e. ∝*T* ^−*n*^ (red line in Figure 1f). On the other hand, the $\sigma_{ba}^{s,\mathrm{SHE}}$ is an intrinsic properties of band structure and independent of scattering, and hence is expected to be weakly dependent on temperature (blue line in Figure 1f) [49]. Therefore, it is possible to disentangle $\sigma_{ba}^{s,\mathrm{SSE}}$ and $\sigma_{ba}^{s,\mathrm{SHE}}$ based on their different temperature dependence and unveil the ultimate nonrelativistic spin polarization of RuO_2_.

SMR is a transport phenomenon associated with charge‐spin conversion and its inverse effect. An in‐plane charge current is first converted to an out‐of‐plane spin current with a polarization ***s***, which is reflected or absorbed by the adjacent ferromagnetic layer depending on the ferromagnetic magnetization ***m***. The reflected spin current, with the intensity proportional to (***m*** · ***s***)^2^, is then converted back to an in‐plane charge current by the inverse effect in addition to the original charge current, resulting in a variation of the overall resistance. Therefore, one can in principle derive the ***s*** of a spin current from the SMR measurement. This technique is especially suitable for investigating the temperature dependence of ***s***, since SMR measurement can be performed with a small DC current and hence avoids the substantial Joule heating which influences the device temperature and enhances the scattering.

**FIGURE 2:**
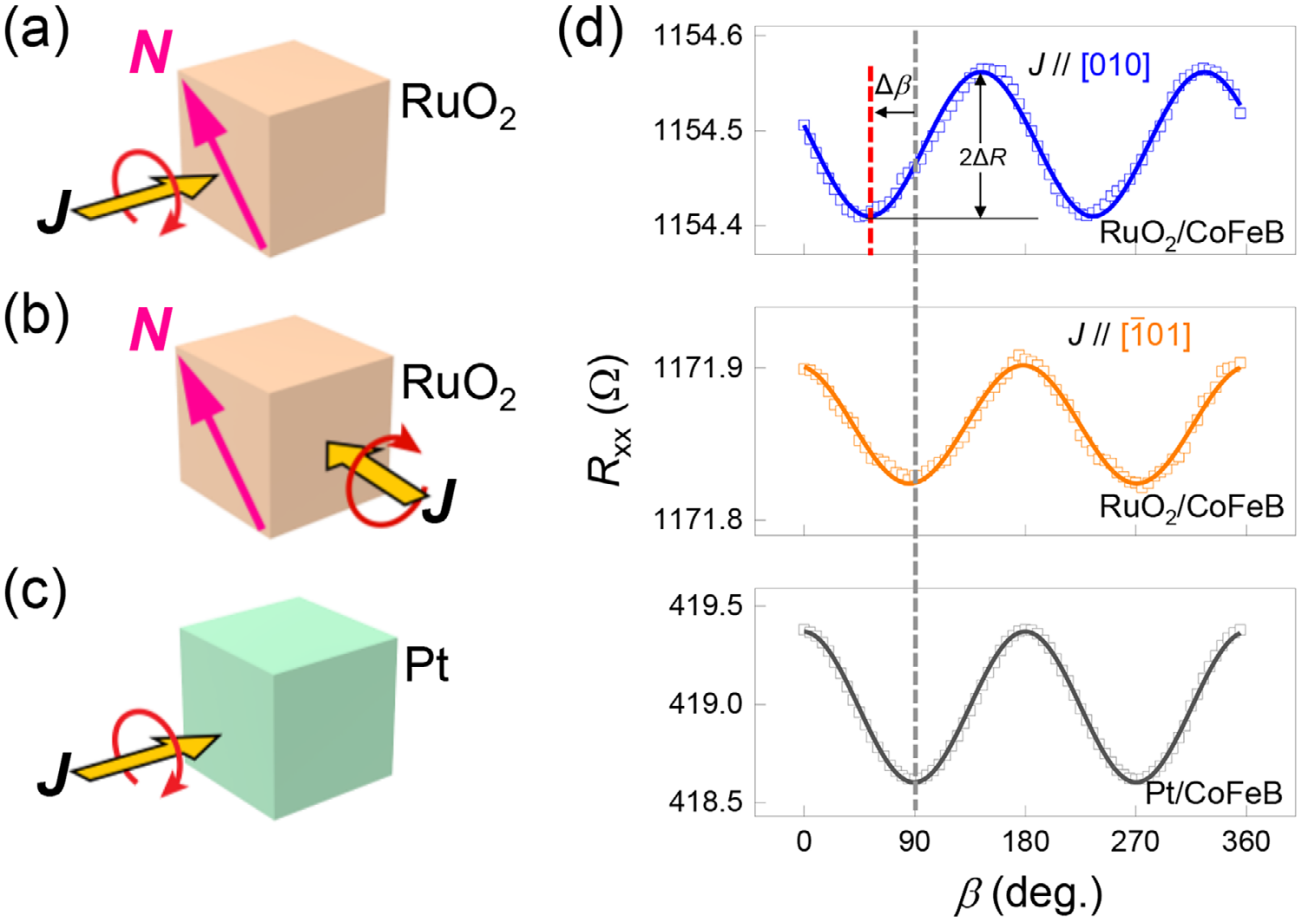
The spin polarization direction of RuO_2_ is extracted by SMR. (a–c) SMR measurement scheme of RuO_2_/CoFeB with *J* // [010] (a), RuO_2_/CoFeB with *J* // [$\bar{1}$01] (b), and Pt/CoFeB (c). (d) SMR in RuO_2_/CoFeB with currents along [010] (blue point) and [$\bar{1}$01] (yellow point) and SMR of Pt/CoFeB (dark point) as a reference sample.

The SMR is measured for RuO_2_/CoFeB and Pt/CoFeB in different geometries (Figure 2a–c). Before measuring SMR, field cooling (FC) was first conducted by applying an 8 T magnetic field in the [001] direction during the cooling process from 400 to 5 K. The fitting blue line in Figure 2d shows the SMR of the RuO_2_/CoFeB sample with *J* // [010] at 5 K. Unlike traditional SMR that majorly follows a Δ*R*cos(2*β*) angular dependence [69], the SMR in Figure 2d (blue line) takes the form Δ*R*cos(2(*β*‐Δ*β*)), where *β* is the angle between the magnetic field and ***z***, Δ*β* is the phase shift, and Δ*R* is the SMR magnitude defined as half the difference between the maximum and the minimum value of resistance *R*(*β*). To eliminate instrumental errors, we deposited Pt/CoFeB on the same Al_2_O_3_ substrate (Note S2) as Figure 1c and measured its SMR to calibrate the angle when measuring SMR of RuO_2_/CoFeB. In Pt/CoFeB, the spin polarization direction is along ***y***, so when ***m***//***y***, the spin current reflected by the FM layer is maximized, leading to the minimum magnetoresistance due to the current induced by the inverse spin Hall effect (ISHE) in Pt, as shown in Figure 2d dark line. When we apply a current along the [010] direction in RuO_2_ (101), the spin current polarization direction has both ***y*** and ***z*** components, the vector sum of their polarization is denoted as ***s***. Therefore, the SMR reaches its minimum when ***m***//***s***, with ***m*** not parallel to ***y***. We also measured SMR with the current along [$\bar{1}$01], where the magnetoresistance is minimal when ***m***//***y***, plotted in Figure 2d (yellow line), indicating no ***z*** component in the spin polarization, consistent with theoretical predictions.

**FIGURE 3:**
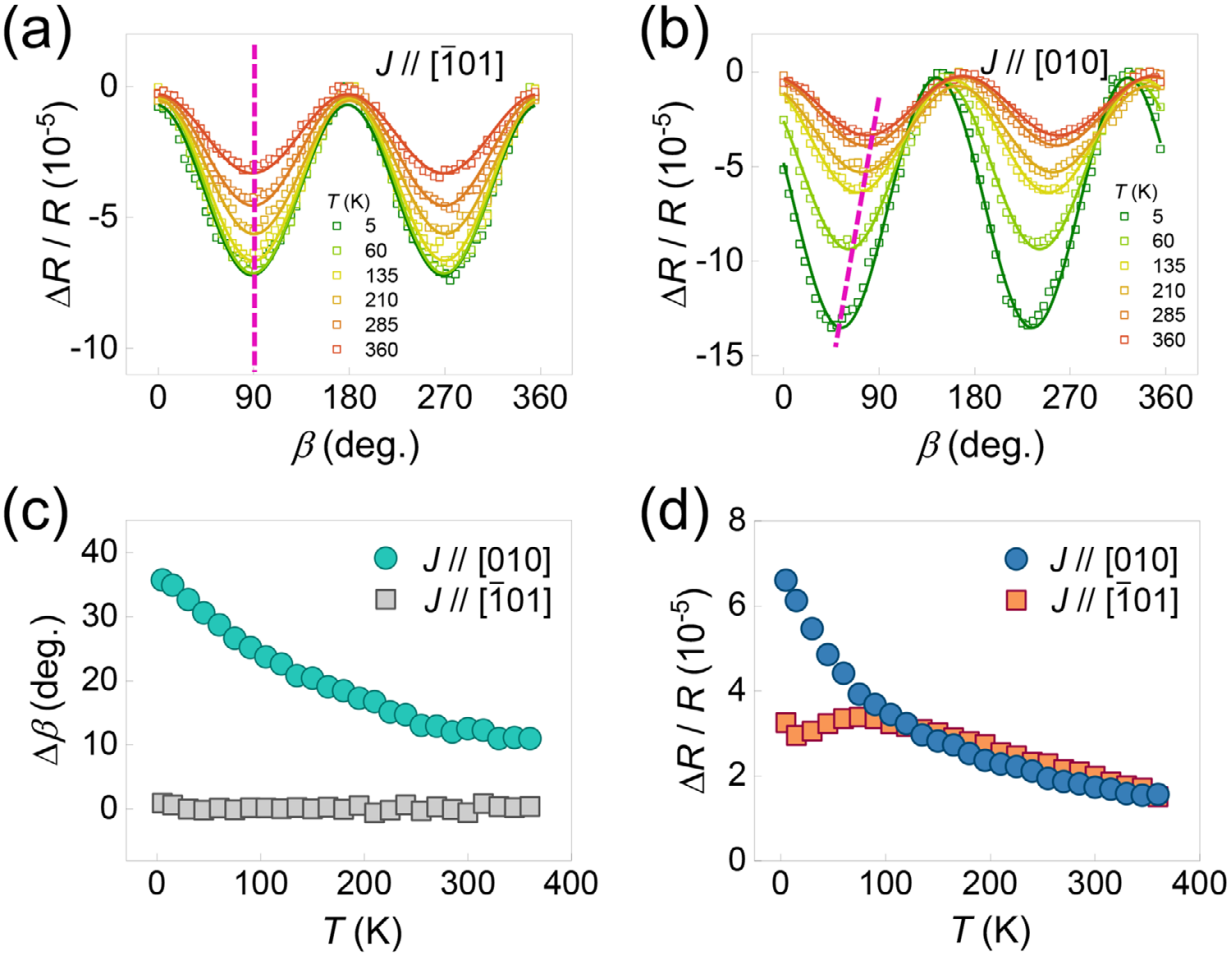
Temperature dependence of SMR. (a,b) The SMR at different temperatures for the current along the [$\bar{1}$01] (a) and [010] (b) directions. (c,d) The temperature dependence of the Δ*β* (c) and Δ*R* (d) derived from the SMR shown in (a) and (b).

Figure 3a,b shows the SMR of RuO_2_/CoFeB at various temperatures with the current along [$\bar{1}$01] and [010] directions. Negligible magnetoresistances of RuO_2_ and CoFeB rule out effects of themselves, such as anomalous planar Hall effect [70], as shown in Note S6. In addition, given the small spin Hall angle of CoFeB [71] and the angular dependence of the spin current generated from CoFeB [72], we can exclude CoFeB as a factor impacting our results (more discussion in Note S7). We find Δ*β* remains zero when the current is along [$\bar{1}$01] (Figure 3a,c). This is because the charge current is parallel to a mirror plane M_[010]_ in this case, which only allows the generation of a ***y***‐polarized spin current. Therefore, it is difficult to reveal the nonrelativistic contribution of the SMR based on the measurements when the current along [$\bar{1}$01]. On the other hand, we find sizable Δ*β* when the current is along [010] (Figure 3b,c), clearly indicating the existence of unconventional spin current polarization. Both Δ*β* and Δ*R* decrease at high temperature (Figure 3c,d), implying the SSE spin conductivity decreases and SHE spin conductivity becomes dominant at high temperature.

**FIGURE 4:**
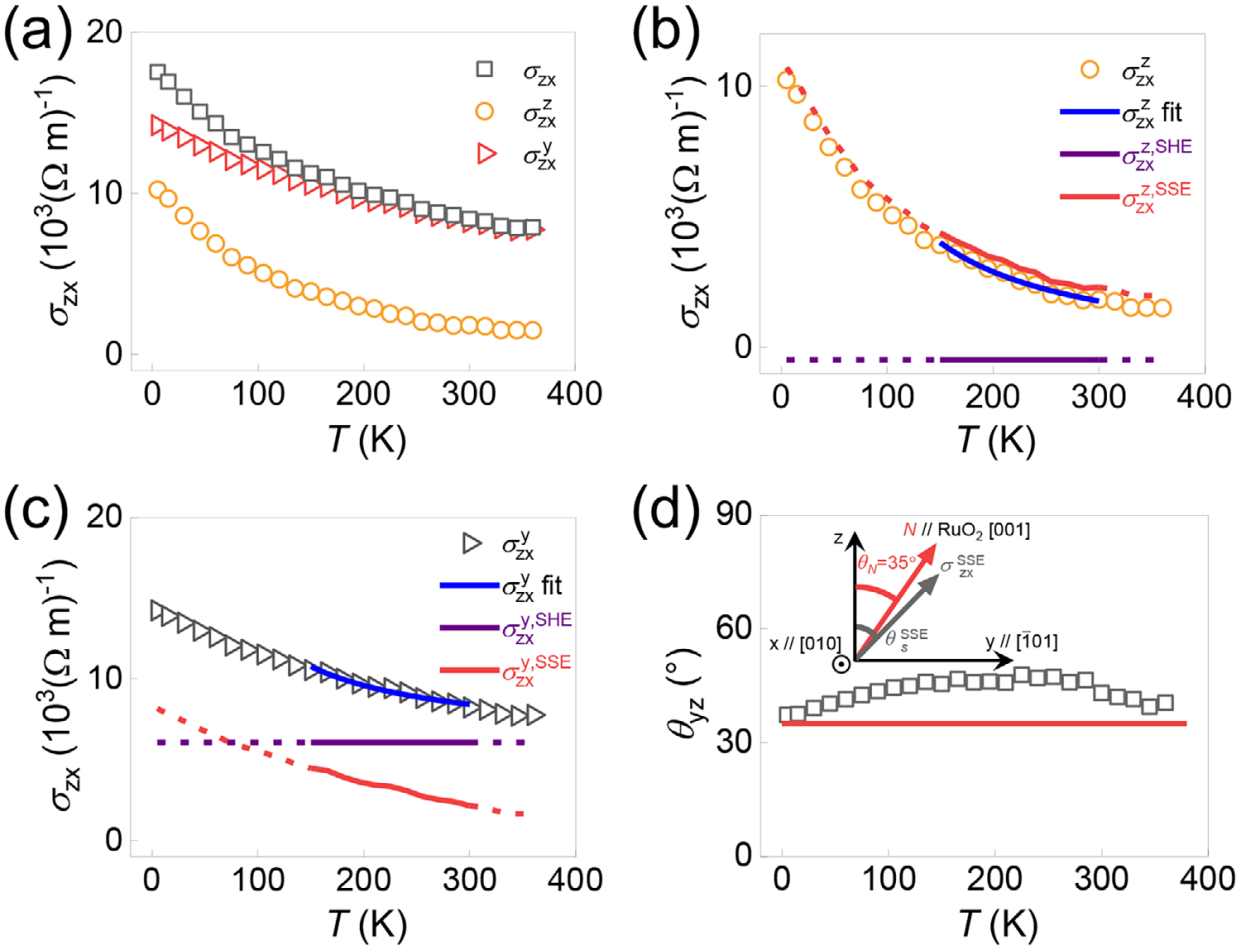
Decomposition of the total spin conductivity into SSE and SHE components via temperature dependence. (a) The total, ***y***‐ and ***z***‐polarized spin conductivity of RuO_2_ derived from SMR measurement. (b,c) The SSE and SHE components of ***z***‐polarized (b) and ***y***‐polarized (c) spin conductivity decomposed by fitting the temperature dependence of the SHC. (d) The SSE spin current polarization indicated by the angle $\theta_{s}^{\mathrm{SSE}}=\arctan\left(\sigma_{zx}^{y,\mathrm{SSE}}/\sigma_{zx}^{z,\mathrm{SSE}}\right)$ as a function of temperature.

Next, we use the SMR results to decompose the SSE and SHE spin conductivities based on their temperature dependence. Assuming zero longitudinal spin absorption and a transparent RuO_2_/CoFeB interface, the SMR results of RuO_2_ fit the following equation [73]:

(1) $$\begin{array}{c} \;\frac{\Delta R}{R}=-\theta_{\mathrm{SH}}^{2}tanh\left(\varepsilon \right)\frac{\lambda_{\mathrm{sf}}}{t_{\mathrm{AF}}}\left[1-\frac{1}{\cosh\left(2\varepsilon \right)}\right] \end{array}$$

where *θ*_SH_ is the spin Hall angle (Figure S3 of Note S3), *λ*_sf_ is the spin diffusion length of 12.2 nm [74], *t*_AF_ is the thickness of RuO_2_, and *ε* = *t*_AF_/2*λ*_sf_. The spin Hall conductivity *σ_zx_* = *θ*_SH_ *σ_xx_* ℏ/2e, where *σ_xx_* = 1/*ρ_xx_*. (*ρ_xx_*‐*T* curve in Figure S4 of Note S3) Since we know the angle Δ*β*, we can decompose the SHC of RuO_2_ into $\sigma_{zx}^{z}=\sigma_{zx}\;\cdot \sin\Delta \beta$ and $\sigma_{zx}^{y}=\sigma_{zx}\;\cdot \cos\Delta \beta$ in the orthogonal ***z*** and ***y*** directions, as shown in Figure 4a. The SSE spin conductivity is proportional to the electron lifetime *τ*, which follows *τ* = *τ*_0_(1/*T*) between 150 and 300 K, where the resistivity of RuO_2_ exhibits a nearly linear temperature dependence (see Figure S6a), making SSE spin conductivity proportional to 1/*T* within this temperature range. Given the weak temperature dependence of SHE spin conductivity (Note S8), we can fit within this temperature range using the relation $\sigma_{zx}^{z}=\;A\left(1/T\right)+B$, as shown by the blue line in Figure 4b, where $\sigma_{zx}^{z,\mathrm{SHE}}=\;B$ is denoted by the purple line in Figure 4b. We then obtain $\sigma_{zx}^{z,\mathrm{SSE}}=\sigma_{zx}^{z}\;-\sigma_{zx}^{z,\mathrm{SHE}}$ over the entire temperature range, shown by the red line in Figure 4b. For $\sigma_{zx}^{y}$, we fit its temperature dependence similarly, as shown in Figure 4c. The resulting fits for both $\sigma_{zx}^{y}$ and $\sigma_{zx}^{z}$ yield R‐squared values exceeding 0.97 (Figure S6b), indicating that this simple model accurately captures the temperature dependence of the spin conductivity in this regime. This excellent agreement suggests that there is no significant anomalous temperature dependence associated with the $\hat{T}$‐even component. We also note that RuO_2_ is an altermagnet with zero net magnetization, its behavior of $\hat{T}$‐even spin conductivity is expected to differ from that in conventional ferromagnets such as SrRuO_3_ [75, 76] which have temperature‐dependent Berry curvature and associated spin transport properties.

We take $\arctan(\sigma_{zx}^{y,\mathrm{SSE}}/\sigma_{zx}^{z,\mathrm{SSE}})$ as $\theta_{s}^{\mathrm{SSE}}$, the angle between the SSE spin current polarization direction and ***z***. At 5 K, $\theta_{s}^{\mathrm{SSE}}=\;\mathrm{arc}\left(\sigma_{zx}^{y,\mathrm{SSE}}/\sigma_{zx}^{z,\mathrm{SSE}}\right)=\;\mathrm{arc}\left[\left(8.2\times 10^{3}\;\Omega^{-1}m^{-1}\right)/\left(10.7\times 10^{3}\;\Omega^{-1}m^{-1}\right)\right]$= 37.3°. $\theta_{s}^{\mathrm{SSE}}$ at different temperatures is plotted in Figure 4d. In (101)‐oriented RuO_2_, considering lattice parameter a = b = 4.59 Å and c = 3.12 Å, the angle between the Néel vector and ***z*** is *θ_N_* = arctan(c/a) = 35°. In Figure 4d it can be seen that within the range of 5 to 360 K, the SSE spin current polarization direction is roughly parallel to the Néel vector.

Our results imply two interesting facts: First, the SSE spin current indeed has a magnetic origin and is polarized along the Néel vector in RuO_2_, despite the nonmagnetic ground state suggested by many recent theoretical and experimental works [65, 66, 67, 68]. This may indicate the unavoidable factors induced by film growth, such as strain, oxygen vacancies, interfacial charge transfer, anti‐site defects, etc., play important roles in stabilizing the magnetism. Indeed, recent work by Qian et al. [77] highlights RuO_2_’s proximity to a quantum phase transition, making its magnetic ground state highly sensitive to perturbations. In Notes S3 and S6, we have shown the exchange bias effect at low temperature and paramagnetism‐altermagnetism transition at 355 K, both of which provide strong validation of the magnetic nature of our RuO_2_ samples. Our result, together with recent observations of spin‐split band structure [35], XMLD [78] and TMR [79] signals, offers solid evidence that magnetism can occur in RuO_2_. Second, although it is generally believed the SOC is a weak effect compared to the magnetic exchange splitting, the SHE‐induced ***y***‐polarized spin current driven by SOC is robust and even much stronger than the SSE‐induced one driven by magnetism, due to the latter being largely suppressed by the scattering at high temperature. This may explain the absence of magnetic signatures in some high‐temperature experiments.

At last, the successful disentangling of SSE and SHE spin currents, which is very difficult using common approaches, clearly indicates the advantages of the method of spin current detection we proposed in this work. We remark that a recent work [80] and our work use different ferromagnetic layers on RuO_2_ (101) films to study the magnetoresistance effect, and the major phenomena (phase shifts and temperature dependences of SMR) observed in the two independent works are well consistent with each other. This unambiguously proves the unconventional SMR effect associated with RuO_2_. Our result of unconventional SMR is applicable not only to altermagnets such as RuO_2_, but also for other unconventional antiferromagnets such as noncollinear antiferromagnets [42, 44, 45, 51, 52]. Moreover, it can effective in unveiling the unconventional polarization of spin currents with other exotic mechanisms, such as the composition gradient [81, 82, 83, 84], symmetry control [85, 86], interface engineering [87], etc. We thus believe the unconventional SMR measurement would be a standard approach for detecting spin current polarizations and thus widely used in the future.

## 3 Conclusion

In conclusion, we report the unconventional SMR generated from nonrelativistic spin currents in RuO_2_, which is capable of accurately detecting spin current polarization and disentangling the SSE and conventional SHE components of a spin current. Based on this approach, we find that the nonrelativistic SSE spin current is polarized along the previously assumed Néel vector. Our results unambiguously reveal the magnetic origin of the nonrelativistic spin current in RuO_2_, and pave a straightforward route to understand the unconventional spin currents that are crucial in spintronics.

## 4 Experimental Section

(101)‐oriented RuO_2_ films are grown by dc magnetron sputtering at 500°C onto Al_2_O_3_ (1$\bar{1}$02) substrates, in a chamber with the base pressure of 5 ×10^−5^ Pa. During deposition, the Ar:O_2_ ratio of the reaction sputtering atmosphere is 4:1, and the working pressure is 0.35 Pa. The deposition rate measured using the atomic force microscope (AFM) is about 0.2 nm/s. After cooling to room temperature, a CoFeB layer is grown by d.c. magnetron sputtering. Then a SiO_2_ capping layer is deposited via radio frequency sputtering. The multilayer structure is Al_2_O_3_ (1$\bar{1}$02)/RuO_2_ (101) (6 nm)/CoFeB (0.9 nm)/SiO_2_ (4 nm).

## Conflicts of Interest

The authors declare no conflicts of interest.

## Supporting information




**Supporting File**: advs74888‐sup‐0001‐SuppMat.docx.

## Data Availability

The data that support the findings of this study are available from the corresponding author upon reasonable request.
