# Supplementary material for: Nonrelativistic Spin Current Generated Unconventional Spin Hall Magnetoresistance in Altermagnetic RuO2
Source: Adv Sci (Weinh). 2026 Mar 18;13(31):e21779. doi: 10.1002/advs.202521779 (PMC13588005; doi:10.1002/advs.202521779)
Supplement: Supplementary file 1 [file ADVS-13-e21779-s001.docx]

**Nonrelativistic spin current generated unconventional spin Hall magnetoresistance in altermagnetic RuO_2_**

Chang Pan,^1,*^ Shuai Hu,^2,*^ Fanli Yang,^3^ Dongchao Yang,^1^ Wei Zhu,^1^ Jianwei Zhang,^1^ Weijia Fan,^1^ Zhong Shi,^1^ Liqing Pan,^3,†^ Shiming Zhou,^1,†^ Xuepeng Qiu^1,†^

^1^ *School of Physics Science and Engineering, Tongji University, Shanghai 200092, China*

^2^ *Institute of Quantum Materials and Devices, School of Electronic and Information Engineering, Tiangong University, Tianjin, 300387, China.*

^3^ *Hubei Engineering Research Center of Weak Magnetic-field Detection, College of Science, China Three Gorges University, Yichang, 443002, China*

^*^These authors contribute equally to this work

^†^e-mail: [lpan@ctgu.edu.cn](mailto:lpan@ctgu.edu.cn), [shiming@tongji.edu.cn](mailto:shiming@tongji.edu.cn) and [xpqiu@tongji.edu.cn](mailto:xpqiu@tongji.edu.cn)

**Table of Contents**

Supplementary Note 1. Sample fabrication

Supplementary Note 2. Angular calibration

Supplementary Note 3. SHC calculation

Supplementary Note 4. SHC decomposition when the current is along [$\bar{\text{1}}$01]

Supplementary Note 5. SMR technique

Supplementary Note 6. Magnetoresistances of RuO_2_ and CoFeB

Supplementary Note 7. Discussion of spin current generated from CoFeB into RuO_2_

Supplementary Note 8. SHCs in Pt/CoFeB and RuO_2_/CoFeB and their temperature dependences

Supplementary Note 9. Doping, epitaxial strain and surface/interface symmetry breaking in RuO_2_

**Supplementary Note 1. Sample fabrication**

(101)-oriented RuO_2_ films are grown by dc magnetron sputtering at 500 °C onto Al_2_O_3_ (1$\bar{\text{1}}$02) substrates, in a chamber with the base pressure of 5 ×10^−5^ Pa. During deposition, the Ar:O_2_ ratio of the reaction sputtering atmosphere is 4:1, and the working pressure is 0.35 Pa. The deposition rate measured by atomic force microscope (AFM) is about 0.2 nm/s. After cooling to room temperature, a CoFeB layer is grown by d.c. magnetron sputtering. Then a SiO_2_ capping layer is deposited via radio frequency sputtering. The multilayer structure is Al_2_O_3_ (1$\bar{\text{1}}$02)/ RuO_2_ (101) (6 nm)/CoFeB (0.9 nm)/SiO_2_ (4 nm).

**FIG. S1:** (**a**) *φ*- and (**b**) 2*θ*-scan spectra of (1$\bar{\text{1}}$02)-oriented Al_2_O_3_ substrate and RuO_2_ (101).

As shown in Fig. S1, *φ*- and 2*θ*-scan spectra of (1$\bar{\text{1}}$02)-oriented Al_2_O_3_ substrate and RuO_2_ (101) confirm the epitaxial growth of RuO_2_ and the absence of in-plane twin crystals on the Al_2_O_3_ (1$\bar{\text{1}}$02) substrate.

We calibrated the growth rate of RuO_2_ to be 0.2 nm/s by growing a thick sample. By controlling the deposition time, we grew RuO_2_ film with the designed thickness. The thickness of this film was fitted to be 6.09 nm using X-ray reflectivity (XRR) characterization. Fig. S2 shows the XRR result and the fitting line.

**FIG. S2:** XRR data and its fitting line.

**Supplementary Note 2. Angular calibration**

Because we need accurate angles for our experiments, we make SMR devices to calibrate the angles. RuO_2_/CoFeB is prepared into double Hall bar devices using standard lithography, and all other regions of RuO_2_/CoFeB on Al_2_O_3_ substrate are removed by Ar ion etching. Then we deposite CoFeB/Pt heterostructures on the bare Al_2_O_3_ substrate near the RuO_2_/CoFeB double Hall bar device as shown in Fig. 1(c). The SMR of CoFeB/Pt is utilized to calibrate the direction of the magnetic field, which is done by setting the direction of the magnetic field at the maximum of the resistance to ***z***.

**Supplementary Note 3. SHC calculation**

We use the SMR results to calculate spin Hall conductivity (SHC). Assuming zero longitudinal spin absorption and a transparent RuO_2_/CoFeB interface, the SMR results of RuO_2_ fit the following equation:

$$\begin{aligned} \frac{\Delta R}{R}=-\theta_{\mathrm{SH}}^{2}tanh(\varepsilon)\frac{\lambda_{\mathrm{sf}}}{t_{\mathrm{AF}}}[1-\frac{1}{cosh(2\varepsilon)}], \#\left( 1 \right) \end{aligned}$$

Where *θ*_SH_ is the spin Hall angle, *λ*_sf_ is the spin diffusion length 12.2 nm, *t*_AF_ is the thickness of RuO_2_, and *ε* = *t*_AF_/2*λ*_sf_.

The calculated *θ*_SH_ is shown in Fig. S3.

**FIG. S3:** Calculated SHA when the current is along [010] (red dot) and [$\bar{\text{1}}$01] (blue dot).

The (101)-oriented RuO_2_ (8 nm) film is prepared as a double Hall bar, with100 μm long and 34 μm wide, and its resistance is measured by the four-points method, and the calculated resistivity is shown in Fig. S4. In the *ρ*-*T* curve with current along [010] or [$\bar{\text{1}}$01], obvious kink has been observed at 355 K, marking the paramagnetism-altermagnetism transition. The transition temperature is also consistent with the Néel temperature of the bulk and thin-film RuO_2_ samples as reported by Berlijn *et al*., Phys. Rev. Lett. 118, 077201 (2017), Zhu *et al*., Phys. Rev. Lett. 122, 017202 (2019), Feng *et al*., Nat. Electron. 5, 735 (2022).

**FIG. S4:** *ρ*-*T* curve of RuO_2_ (8 nm) with current along [010] (**a**) and [$\bar{\text{1}}$01] (**b**).

Spin Hall conductivity *σ*_zx_ = *θ*_SH_*σ*_xx_ℏ/2e, where *σ*_xx_ = 1/*ρ*_xx_.

The SHC when the current is in the [010] direction is shown in the red dot in Fig. 4(a), and the SHC when the current is in the [$\bar{\text{1}}$01] direction is shown as the blue dot in Fig. S5.

**Supplementary Note 4. SHC decomposition when the current is along [**$\bar{\text{1}}$**01]**

**FIG. S5:** SHC when the current is along [$\bar{\text{1}}$01] (blue dot), and the decomposed SSE (red line) and SHE (purple line) spin conductivities.

The dark dots in Fig. 3(c) indicate that the Δ*β* is 0 when the current is along [$\bar{\text{1}}$01], which suggests that the direction of its spin polarization is along ***y***. Next, we use the scheme of decomposing the SSE and SHE spin currents in the main text to decompose the SHC when the current is along [$\bar{\text{1}}$01], as shown in Fig. S5. It can be seen that the total SHC has a clear temperature dependence due to the SSE spin conductivity.

**Supplementary Note 5. SMR technique**

As discussed in the manuscript, in a collinear magnetic metal within the nonrelativistic limit, the charge conductivity and the SSE spin conductivity are closely related and are expected to exhibit the same temperature dependence, typically following a power-law behavior $\propto T^{-n}$. Since the temperature dependence of the resistivity (or conductivity) of RuO_2_ can be readily measured, we use this known dependence to isolate the SSE-induced component from the total spin conductivity.

For simplicity, we focus on the temperature range from 150 K to 300 K, where the resistivity of RuO_2_ exhibits a nearly linear temperature dependence (see Fig. S6(a)). Accordingly, we fit the total spin conductivity using the relation $\sigma_{zx}^{i}=AT^{-1}+B$. The resulting fits for both $\sigma_{zx}^{y}$ and $\sigma_{zx}^{z}$ yield R-squared values exceeding 0.97 (Fig. S6(b)), indicating that this simple model accurately captures the temperature dependence of the spin conductivity in this regime. This excellent agreement suggests that there is no significant anomalous temperature dependence associated with the SHE component.

**FIG. S6:** **(a)** Temperature dependence of 1/conductivity in RuO_2_ (101) and its fitting. **(b)** ***z***- and ***y***-polarized SHCs of RuO_2_ derived from SMR measurement, and their fitting lines.

Based on this analysis, we assign the $AT^{-1}$ term to the SSE spin conductivity and identify the nearly constant $B$ term as the dominant SHE contribution. This implies that the spin Hall conductivity in RuO_2_ has a significantly weaker temperature dependence compared to the SSE part. This behavior is consistent with the intrinsic nature of the SHE signal, which originates from the Berry curvature in the band structure and is largely insensitive to scattering processes.

We also note that RuO_2_ is an altermagnet with zero net magnetization, which distinguishes it fundamentally from ferromagnetic systems such as SrRuO_3_. Therefore, the behavior of spin Hall conductivity of RuO_2_ are expected to differ from those in conventional ferromagnets.

Due to its altermagnetic symmetry, RuO_2_ (001) is unique in that it exhibits no SSE spin conductivity, making it an ideal reference system for isolating and characterizing the temperature dependence of the spin Hall conductivity. While we acknowledge that the magnitude of the spin Hall conductivity may differ quantitatively between RuO_2_ (001) and (101) orientations due to anisotropy in the spin-orbit coupling and spin Berry curvature, we argue that their temperature dependence is expected to be similar.

**FIG. S7.** Temperature dependence of 1/conductivity in RuO_2_ (101) and RuO_2_ (001)

This expectation is supported by experimental observations: both RuO_2_ (001) and (101) exhibit similar temperature dependences of electrical resistivity (see Fig. S7), consistent with previous reports (e.g., Feng *et al*., Nat. Electron. 5, 735 (2022)). Since resistivity reflects the underlying electronic scattering and band structure evolution with temperature, its similar behavior across orientations suggests that the dominant mechanisms governing charge and spin transport are qualitatively unchanged. Therefore, we do not expect a fundamental difference in the thermal behavior of the spin Hall conductivity between these two orientations. Accordingly, the (001) measurement provides a reliable basis for inferring the temperature-independent nature of the SHE contribution in RuO_2_ more generally.

Various detection methods have been developed to analyze spin currents using ferromagnetic layers. Periodic spin currents cause magnetic moment oscillations, leading to anisotropic magnetoresistance (AMR) rectification and second-harmonic Hall effects. Traditional methods like spin-torque ferromagnetic resonance (ST-FMR), second-harmonic Hall, and anomalous Hall effect loop-shift can effectively provide the equivalent field of the interaction between spin currents and magnetic moments, allowing the determination of out-of-plane spin polarization. However, direct measurement techniques for spin polarization direction are still lacking. Bilinear magnetoelectric resistance (BMER) can accurately provide spin polarization direction but is limited to layered materials and two-dimensional systems. A more general measurement method for spin polarization direction would greatly aid the study of out-of-plane spin polarization.

We compare here ST-FMR, 2nd-harmonic Hall effects, and SMR used in this paper. Of these three methods, 2nd-harmonic Hall and ST-FMR are based on signals from magnetic moment oscillations to characterize the torque magnitude and hence the SHA, while SMR is a new intrinsic magnetoresistive effect. So, both ST-FMR and 2nd-harmonic Hall require AC or high-frequency current input, while SMR only requires DC. Also, 2nd-harmonic Hall and ST-FMR require a large enough current to bring about significant magnetic moment oscillations. However, SMR does not require a large current. ST-FMR can separate the signals from different magnetic layers (if there are two or more magnetic layers in the heterostructure) because the resonance fields of different magnetic layers are not the same.

In short, the advantages of SMR are:

a. SMR can use smaller current densities, avoiding the degradation of ferromagnetic layers due to electromigration and Joule heating. SMR does not require resonant microwave fields and alternating current as well as lock-in amplifiers, which reduces the complexity of the experimental setup. The device structure of SMR is simpler compared to ST-FMR.

b. In ST-FMR and 2nd-harmonic Hall measurements, the device geometry has the potential to induce SOT-like signals, which affects the fitted SOT effective field.

c. In addition, 2nd-harmonic Hall (in-plane magnetic anisotropy system) and ST-FMR cannot fit the field-like torque of ***z*** spin, which may affect the calculated spin Hall conductivity.

The disadvantages of SMR are:

a. Our current technique can only measure ***y*** and ***z*** spin, if we want to measure ***x*** spin, we need to develop a process to grow ferromagnetic insulators or exfoliate van der Waals ferromagnetic insulators first.

b. SMR measurement requires a large magnetic field to make ***m***//*H*, and may not be able to measure systems consisting of ferromagnetic spin sources.

In the prior studies (Wang *et al*., Phys. Rev. Lett. 133, 046701(2024), Liao *et al*., Phys. Rev. Lett. 133, 056701(2024), Bose *et al*., Nat. Electron. 5, 267 (2022)), Wang *et al*. used ST-FMR and spin pumping techniques to prove that the spin Hall effect is dominant in RuO_2_, and Liao *et al*. used thermoelectric techniques to separate the different contributions of spin Hall effect, and Bose *et al*. obtained the polarization direction of SSE spin current at 300 K by fitting the angular dependence of SHC. Our work and these studies all focus on the contributions of different mechanisms to the spin current in RuO_2_. The difference lies in that we quantitatively distinguished the SOC-induced and symmetry-driven contributions using temperature-dependent relationships from 5 K ~ 360 K. The other works only performed the disentanglement of SHC at one specific temperature by using other experimental methods. The unconventional SMR reported in this work provides an innovative, simple and straightforward method to study the intriguing spin transport physics and explore new spintronic materials.

Table S1. Comparison of disentanglement of SOC-induced and symmetry-driven contributions

| Sample  structure | Experimental  method(s) | Temperature for  disentangling SHC | Whether  quantitative | Ref. |
| --- | --- | --- | --- | --- |
| RuO_2_/CoFeB | SMR | 5 K ~ 360 K | Yes | This work |
| RuO_2_/Py | ST-FMR | 300 K | Yes | Bose *et al*., Nat. Electron. 5, 267 (2022) |
| RuO_2_/YIG | Spin seebeck effect | N.A. | Yes | Liao *et al*., Phys. Rev. Lett. 133, 056701(2024) |
| RuO_2_/Py | Spin pumping and ST-FMR | N.A. | No | Wang *et al*., Phys. Rev. Lett. 133, 046701(2024) |

**Supplementary Note 6. Magnetoresistances of RuO_2_ and CoFeB**

In order to exclude the magnetoresistance of RuO_2_ and CoFeB themselves, we prepare two samples, RuO_2_ (8 nm) and RuO_2_ (6 nm)/SiO_2_ (6 nm)/CoFeB (0.9 nm)/SiO_2_ (4 nm). Using the same measurement scheme as in the main text, a current of 100 μA is applied in the [010] direction, and a 4 T magnetic field is scanned in the ***y-z*** plane. The magnetoresistances measured at different temperatures are shown in Fig. S8, where the range of the ***y***-axis is the same as that of Fig. 3(b). It can be seen that the magnetoresistance of RuO_2_ itself is negligible with respect to the SMR of RuO_2_/CoFeB. By inserting SiO_2_ between RuO_2_ and CoFeB, the transmission of spin current to the ferromagnetic layer is isolated. The magnetoresistance at this time is also negligible, as shown in Fig. S8c, indicating that we can ignore the contribution of CoFeB itself to the SMR. The above results corroborate that the magnetoresistance of RuO_2_/CoFeB originates from the reflection of spin current by CoFeB and the subsequent spin-charge conversion.

**FIG. S8:** **(a,b,c)** *β*-scan ADMR of RuO_2_ (101) (6 nm)/ CoFeB (0.9 nm), RuO_2_ (101) (8 nm) and RuO_2_ (101) (6 nm)/SiO_2_ (6 nm)/CoFeB (0.9 nm) device..

We have measured all the three angle dependence measurements, namely, the *α*, *β* and *γ*-scan SMR with the current applied along the [010] direction. The results are shown in Fig. S9(a). Figs. S9(b-d) further present the magnetoresistances at different temperatures. As can be seen, only the *β*-scan SMR exhibits a phase shift with temperature, whereas the *α*- and *γ*-scan SMRs maintain phase alignment with the standard axis and show no phase shift. These results are well in-line with the physics schematic drawn in Fig. 1 and further validate the central role of Néel vector: In the *α*-scan SMR, the RuO_2_ (101) only projects its Néel vector along ***y***-axis and thus no phase shift should be observed relative to the Cartesian coordinate axes when the magnetization is rotated in the ***x*-*y*** plane. Similarly, in the *γ*-scan SMR, the RuO_2_ (101) only projects its Néel-vector along ***z***-axis and thus no phase shift should be observed relative to the Cartesian coordinate axes when the magnetization is rotated in the ***x*-*z*** plane.

**FIG. S9:** **(a)** *α*, *γ*, *β*-scan SMR of (101)-oriented RuO_2_ (6 nm)/CoFeB with a 4 T magnetic field at 50 K with the current along [010]. **(b,c,d)** *β* (b), *α* (c), *γ* (d)-scan SMR at different temperatures.

A strong magnetic field can cause RuO_2_ spin flop, allowing the Néel vector to rotate to a direction perpendicular to the magnetic field. However, after the external magnetic field is removed, the Néel vector relaxes, and its final orientation is determined by factors such as the dead layer, interface, and defects, rather than the external magnetic field. Therefore, the change in sign of SSE spin conductivity caused by the Néel vector switching can not be observed.

Considering that the easy axis of RuO_2_ is along [001], the Néel vector of as-prepared RuO_2_ may be partially along [001] and remaining part along [00], and the antiferromagnetic domains that cancel each other out weaken the SSE spin current. After field cooling, the Néel vectors align, and spin Hall magnetoresistance (SMR) angular shift (Δ*β*) is enhanced.

Square dot in Fig. S10 shows the SMR curve at 10 K of the sample in the initial state before field cooling. And 5 sets of field-cooling (FC) experiments have been performed as suggested. The FC conditions are listed below:

1. FC1: 400 K to 5 K with 8 T // [001].
2. FC2: 400 K to 5 K with -8 T // [001].
3. FC3: 400 K to 5 K with 8 T // [100].
4. FC4: 400 K to 5 K with -8 T // [100].
5. FC5: 523 K to RT with 0.8 T // [010].

After each set of FC, the SMR was measured. The SMR curves are shown in Fig. S10(a), indicating that after the first FC, Δ*β* increased compared to the initial state. Fig. S10(b) confirms that at different temperatures, Δ*β* after the first FC is greater than the initial state. However, no visible change of SMR has been observed after subsequent FC2 ~ FC5. Fig. S10(b) further shows there is no variation of phase shift Δ*β* for different FC.

**FIG. S10:** **(a)** SMR at 10 K of the sample in the initial state and SMR at 5 K after 5 sets of FC. **(b)** Summary of Δ*β* after FC.

To unambiguously prove the $\hat{T}$-odd nature of the observed RuO_2_ spin current, we have performed additional SMR measurements with different magnetic field. As shown in Fig. S11(a), a clear variation of phase shift of the SMR with different magnetic field for RuO_2_ (101)/CoFeB has been identified, identifying the magnetic origin of the spin current. On the other hand, SMR in Pt/CoFeB and RuO_2_(001)/CoFeB do not show such a phase shift variation shown in Fig. S11(b,c). The former is because Pt can support only the conventional spin current introduced by SOC. For the latter, although the magnetic field may influence Néel vector or domain structure in RuO_2_(001) film, it will not influence phase shift of SMR since the SSE spin current due to spin splitting effect only occurs in the in-plane directions, and hence only conventional spin current by SOC is responsible for SMR in this case.

**FIG. S11:** *β*-scan SMR for RuO_2_ (101)/CoFeB (**a**), Pt/CoFeB (**b**) and RuO_2_ (001)/CoFeB (**c**) with 1 T to 8 T external magnetic field at 5 K. Noted that the fitting of Δ*β* is performed for the full angular range 0° ~ 360° (same with Fig. 2) while (**a-c**) only show partial angular range to more clearly visualize the phase shift of SMR.

We calculated the SHC of a reference sample, Pt/CoFeB, at room temperature. The SMR magnitude (Fig. S12) is 0.11%, yielding a SHA and SHC for Pt of 0.076 and 2.0×10^5^ (Ω m)^-1^, respectively, roughly similar to literature values (Liu *et al*., Phys. Rev. Lett. 106, 036601 (2011)). This validates the reliability of our SMR-like method for measuring SHC in RuO_2_. Using this technique, we determined the SHC of RuO_2_ at room temperature to be 0.84×10^4^ (Ω m)^-1^.

**FIG. S12:** SMR of reference sample, Pt/CoFeB, at 300 K.

We have also measured the anisotropic magnetoresistance (AMR) of RuO_2_/CoFeB heterostructures at 5 K after a field cooling (FC) process, in which a ±8 T magnetic field was constantly applied during the temperature cooling from 380 K to 5 K, as shown in Fig. S13. To more clearly visualize the possible exchange bias effect, we have plotted the mirrored AMR curve with respect to the ***y***-axis for comparison. As can be seen from Fig. S13(a,b), we do not observe the exchange bias shift in the RuO_2_/CoFeB heterostructures at 5 K. The absence of exchange bias effect in RuO_2_/CoFeB could be related to the amorphous crystalline structure of CoFeB which hinders the establishment of good exchange coupled interface (Fecioru-Morariu *et al*., J. Appl. Phys.102, 053911(2007) and Fujita *et al*., J. Appl. Phys. 81, 4909 (1997)). Therefore, we have further replaced CoFeB with permalloy to examine the exchange bias effect. A distinct exchange bias effect can be clearly observed in Fig. S13(d), which is not only reflected in the loop shift of -8 Oe but also by the asymmetric loop shape of the ascending and the descending AMR sub-branches. Furthermore, by reversing the FC magnetic field to -8 T, an opposite exchange bias emerges with a loop shift of +2 Oe and a reversed loop asymmetry (Fig. S13(e)). While further investigations are necessary to elucidate the specific FC and measurement configurations required for clearly observing the exchange bias effect, the demonstration of exchange bias effect in our RuO_2_ based heterostructure unambiguously proves the magnetic nature of RuO_2_ and provides strong support of this study.

**FIG. S13:** AMR of RuO_2_ (101) (6 nm)/CoFeB (0.9 nm) for *H* // [$\bar{\text{1}}$01] (**a**) and *H* // [010] (**b**) after 8 T FC, and of RuO_2_ (101) (10 nm)/Py (4 nm) for *H* // [$\bar{\text{1}}$01] (**c**) and *H* // [010] (**d**,**e**) at 5 K with *J* // [$\bar{\text{1}}$01]. In (c,d) and (e), 8 T and -8 T FC were performed, respectively. The sweep magnetic field has been calibrated by the reference Pt/CoFeB sample which was measured simultaneously to subtract the residual magnetic field.

To further confirm the exchange bias effect, we have first examined the exchange bias effect with different relative orientation between *H*_FC_ and *H*. A field cooling (FC) with an 8 T *H*_FC_ // [$\bar{\text{1}}$01] from 400 K down to 5 K was performed. As can be seen in Fig. S14, a large exchange bias field of -28 Oe for *H* // [010] and -15 Oe for *H* // [$\bar{\text{1}}$01] have been clearly observed. Please noted that this time an improved FC process from 400 K (stabilized for a duration of 10 mins) to 5 K has been performed as compared to 380 K (stabilized for a duration of only 2 secs) to 5 K for prior results, leading to a significantly larger exchange bias effect. The larger exchange bias field for *H* ⊥ *H*_FC_ as compared to *H* // *H*_FC_ may relate to the spin-reorientation (spin-flop) under the large *H*_FC_, which has been also reported recently for altermagnetic MnTe (Betancourt *et al*., npj spintronics 2, 45 (2024)).

Second, we have measured the exchange bias effect from 2.3 K to 100 K (Fig. S15). *H*_EB_ decreases to zero above 8 K, while the enlargement of *H*_c_ (which is another prominent feature of interfacial exchange coupling) persists until 25 K.

Third, we have measured the training effect by measuring 4 consecutive AMR curves at 5 K (Fig. S16). The second AMR curve exhibits much smaller exchange bias effect and *H*_EB_ vanishes and *H*_c_ becomes constant after the measurements of the first two curves, which provides strong validation for the existence of exchange bias effect.

**FIG. S14:** AMR curves of RuO_2_ (101) (10 nm)/Py (4 nm) measured for *H* // [010] (**a**) and *H* // [$\bar{\text{1}}$01] (**b**) with *J* applied along [$\bar{\text{1}}$01]. Before each measurement, a FC with an 8 T magnetic field // [$\bar{\text{1}}$01] from 400 K down to the measurement temperature (5 K) was performed. Noted that the residual magnetic field of the superconductor magnet has been calibrated by a reference Py/Ta sample which was measured simultaneously with the RuO_2_/Py sample.

**FIG. S15:** Temperature dependence of exchange bias. **(a-b)** AMR of RuO_2_ (101) (10 nm)/Py (4 nm) for *H* // [010] at 2.3 K and 8 K with *J* // [$\bar{\text{1}}$01]. **(c-d)** The temperature dependences of coercivity (*H*_c_) and exchange bias field (*H*_EB_). An 8 T FC with *H*_FC_ // [$\bar{\text{1}}$01] down from 400 K was performed before each measurement. The *H* has been calibrated by a reference sample similar to Fig. 1 and Fig. S14.

**FIG. S16:** Training effect of exchange bias. **(a)** Conecutive measurements of AMR curves of RuO_2_ (101) (10 nm)/Py (4 nm) for *H* // [010] with *J* // [$\bar{\text{1}}$01] at 5 K. An 8 T FC with *H*_FC_ // [$\bar{\text{1}}$01] down from 400 K was performed before the consecutive sweeps of *H*. **(b)** *H*_c_ and |*H*_EB_| versus sweep number. The *H* has been calibrated by a reference sample similar to Fig. 1 and Fig. S14.

During the systematic transport measurements, we are able to precisely calibrate the residual magnetic field from the on-chip reference device (see Fig. 1) to accurately measure the exchange bias effect. The magnetic nature has been now unambiguously proved by the four distinct features of exchange bias (Qiu *et al*., Phys. Rev. Lett. 101, 147207 (2008)) in the RuO_2_/Py:

(1) Prominent exchange bias field;

(2) Pronounced enhancement of coercivity;

(3) Asymmetric magnetization reversal behavior;

(4) Characteristic training effect.

These mutually consistent phenomena collectively provide solid evidence of magnetism in RuO_2_.

**Supplementary Note 7. Discussion of spin current generated from CoFeB into RuO_2_**

First, the spin Hall angle of CoFeB has been reported to be very small (~0.007 for 5 nm CoFeB in Yang *et al*., Phys. Rev. Applied 13, 034072 (2020)), which serves as the upper limit of our 0.9 nm CoFeB after considering the approximately linear scaling of ferromagnet spin Hall angle with thickness (Hayashi *et al.*, Commun. Phys. 6, 32 (2023)). Therefore, the spin Hall angle of CoFeB is too small to account for the spin Hall angle of our sample which is ~0.02 as shown in Fig. S3.

Second, the angular dependence of the spin current generated from CoFeB allows us to further exclude its effect on our SMR results. Ferromagnetic metals (FM) are capable of generating spin currents through spin Hall effect (SHE), anomalous Hall effect (AHE) (Taniguchi *et al*., Phys. Rev. Appl. 3, 044001 (2015)) and planar Hall effect (PHE) (Safranski *et al*., Nat. Nanotechnol. 14, 27-30 (2019)). Other mechanisms such as spin–orbit filtering (Amin *et al*., Phys. Rev. Lett. 121, 136805 (2018)), spin anisotropic magnetoresistance effect (Taniguchi *et al*., Phys. Rev. Appl. 3, 044001 (2015)). spin swapping (Lifshits *et al*., Phys. Rev. Lett. 103, 186601 (2009)) and interfacial spin–orbit precession (Amin *et al*., Phys. Rev. Lett. 121, 136805 (2018)) may also play a role. However, these effects accommodate distinct different angular dependence as compared to our RuO_2_ results as analyze below:

For FM-associated spin currents, for *J* // ***x***, the spin polarization ***σ*** takes the general form of:

***σ***=*σ_y_****y***+*σ_m_****m***+*σ_m_*_×_*_y_*(***m***×***y***)

where *σ_y_*, *σ_m_* and *σ_m_*_×_*_y_* are the charge-to-spin conversion efficiencies for the corresponding polarization components (Ryu *et al*., Nat. Electron. 5, 217 (2022)). Each component represents specific charge-to-spin conversion mechanisms: ***y*** polarization corresponds to the SHE and interfacial spin–orbit filtering, ***m*** polarization corresponds to the spin anomalous Hall effect and spin anisotropic magnetoresistance effect, and ***m***×***y*** polarization corresponds to spin swapping and interfacial spin–orbit precession.

Briefly, the ***y*** polarization is not correlated with the ***m*** direction. The ***m*** and ***m***×***y*** polarizations changes with the ***m*** direction with a period of 2π. Since our SMR period is π, the spin current generated from CoFeB does not make notable contribution in present study.

As for PHE, it depends on the angle between the magnetization and the current (Goldberg *et al*., Phys. Rev. 94, 1121 (1954), Seemann *et al*., Phys. Rev. Lett. 107, 086603 (2011)). In our *β*-scan SMR, the magnetization is rotated in the ***y***-***z*** plane, and the current is always perpendicular to the CoFeB magnetization. Consequently, the PHE keeps invariant and does not contribute to the observed results.

**Supplementary Note 8. SHCs in Pt/CoFeB and RuO_2_/CoFeB and their temperature dependences**

To validate the schematics of Fig. 1(f) and achieve self-consistency of the conclusion, we have measured the temperature dependence of SHC in our RuO_2_/CoFeB and Pt/CoFeB devices. The Pt/CoFeB device represents the standard example hosting $\sigma_{s}^{even}$-domainated SHC. In RuO_2_ (001)/CoFeB, the altermagnetic spin-splitting mechanism does not generate spin current flowing vertically toward the CoFeB layer, and hence only the conventional SHE generated $\sigma_{s}^{even}$ is presented in our measurements. The results are shown in Fig. S17. As compared to the RuO_2_ (101) /CoFeB, both the Pt/CoFeB and RuO_2_ (001)/CoFeB exhibit much weaker temperature dependent SHC, which provide strong validation to the temperature dependence of $\sigma_{s}^{even}$ and $\sigma_{s}^{odd}$ that schematically drawn in Fig. 1(f).

**FIG. S17:** **(a)** *β*-scan SMR of Pt (5 nm)/CoFeB (3 nm) device. **(b)** The temperature dependence of the SMR/SMR_300 K_ (SMR = Δ*R*/*R*) for Pt/CoFeB (gray square) and RuO_2_ (101)/CoFeB (blue circle). **(c)** The temperature dependence of the SHC/SHC_300 K_ for Pt/CoFeB (gray hollow square) and ***z***-polarized SHC/SHC_300 K_ for RuO_2_ (101)/CoFeB (yellow hollow circle). **(d)** *β*-scan SMR of RuO_2_ (001) (6 nm)/CoFeB (0.9 nm) device. **(e)** The temperature dependence of the SMR/SMR_300 K_ for RuO_2_ (001)/CoFeB (magenta square) and RuO_2_ (101)/CoFeB (blue circle). **(f)** The temperature dependence of the SHC/SHC_300 K_ for RuO_2_ (001)/CoFeB (magenta hollow square) and RuO_2_ (101)/CoFeB (blue hollow circle).

**Supplementary Note 9. Doping, epitaxial strain and surface/interface symmetry breaking in RuO_2_**

We remark a recent work by Qian *et al*. (Phys. Rev. B 111, 174425 (2025)) highlights RuO_2_’s proximity to a quantum phase transition, making its magnetic ground state highly sensitive to perturbations. The difference of our sample as compared to the ones reported non-magnetic nature of RuO_2_ (ref. 65 ~ 68) may relate to the different sample crystallinity, the fabrication method, and the sample form (bulk crystal vs. thin film) *etc*. The factors mentioned by the reviewer, such as doping, epitaxial strain and surface/interface symmetry breaking, are discussed and clarified below:

a． Doping: Here, reaction sputtering of high purity Ru target with Ar/O_2_ mixed gas is performed to grow RuO_2_ thin films under high vacuum without other elemental doping. Our XRD and TEM results well support the single crystallinity of RuO_2_ thin films.
b． Epitaxial strain: Heteroepitaxial growth introduces significant lattice stress into RuO_2_ thin films, with the fundamental mechanism stemming from lattice mismatch between the film and substrate. In this study, 6 nm RuO_2_ (101) epitaxial layer deposited on Al_2_O_3_ (102) substrate exhibits a lattice mismatch of 6.8% along [01] and -5.3% along [010] direction (substrate parameters: a=4.75 Å and $\sqrt{3a^{2}+c^{2}}/3$=5.12 Å, bulk RuO_2_: a=4.50 Å and $\sqrt{a^{2}+c^{2}}$=5.47 Å). Jeong *et al*., arXiv:2405.05838 confirms that 17 nm RuO_2_(110) sample maintains obvious epitaxial strain, implying that our 6-nm-thick RuO_2_ exhibits coherent strain throughout the entire layer. And quantitative analysis of the (101) Bragg peak at 2θ = 35.22° (Fig. S1) reveals a 0.34% contraction in out-of-plane interplanar spacing relative to bulk RuO_2_, indicative of compressive strain along the surface normal direction.
c． Surface/interface symmetry breaking: When the film thickness is reduced to the nanometer scale, symmetry breaking at the surface and interface significantly regulates the macroscopic properties of the material, which may influence the RuO_2_ magnetic state.

As analysed above, our RuO_2_ sample has a strain from the substrate and symmetry breaking at the interface, which might influence the magnetic state of RuO_2_(Brahimi *et al*., arXiv:2412.15377 and Qian *et al*., Phys. Rev. B 111, 174425 (2025)).
